# The effects of breeding young bulls and cows *in vitro*

**DOI:** 10.1590/1984-3143-AR2025-0047

**Published:** 2025-08-11

**Authors:** Marc Andre Sirard

**Affiliations:** 1 Centre de Recherche en Reproduction, Développement et Santé Intergénérationnelle – CRDSI, Université Laval, Québec, Canada

**Keywords:** epigenetic, DNA methylation, gametes, embryos, age

## Abstract

In the bovine dairy sector, the pursuit of rapid genetic advancement has prompted the adoption of increasingly younger parental figures for both males and females. While physiological limitations and access to gametes impose certain restrictions, the impact of age on gamete quality remains crucial yet poorly understood. We propose that the age effect encompasses environmental factors, which include the metabolic state of the parents and the conditions surrounding gametes and embryos within the reproductive tracts of both sexes. Emerging evidence indicates that this environment significantly influences not only the functionality of gametes and early embryos but also the future phenotype of the offspring. Recent research utilizing transcriptomic and epigenetic molecular analyses in bovine models has demonstrated that the age of both females and males gamete donors, can alter gene expression and programming within the embryo in a similar way that metabolic post partum conditions can. This embryo adaptation to parent’s age is similarly noted in variations related to different culture conditions and the in vitro fertilization (IVF) process. A common outcome from these circumstances is the development of embryos operating in “economy” mode, where translation, cell division, and ATP production are diminished, seemingly as an anticipated adaptation to environmental conditions. Furthermore, new epidemiological studies have shown that these alterations can lead to distinct phenotypes, particularly in animals conceived through IVF, underscoring the long-term consequences that may unfold later in their lives.

## The mechanics of epigenetics

The notion that phenotypic changes induced by environmental factors can be inherited across generations has been recognized for centuries. However, it is only in recent decades, with advancements in epigenetics, that we have gained valuable insights into the mechanisms through which the environment influences an organism's phenotype.

Although the intergenerational transmission of epigenetic marks is a well-established phenomenon, the transgenerational transmission particularly in protein-coding regions remains a matter of debate in mammalian species (Khatib et al., 2024). The same author published very supportive data obtained in the sheep that do support the transgenerational legacy (Braz et al., 2022)

Epigenetics encompasses three primary components: DNA methylation, histone modifications, and non-coding RNAs. DNA methylation is long-term and not easily reversible, whereas histone modifications represent short-term changes that are more readily reversible. Non-coding RNAs exhibit mid-term effects and are also reversible. Unlike static genetic information, epigenetic factors are susceptible to influence from environmental changes.

Research has indicated that these epigenetic alterations can be inherited both trans-generationally and intergenerationally, impacting the performances of subsequent generations. This highlights the importance of understanding epigenetic mechanisms, particularly in the context of dairy cattle, where both environmental conditions and genetic selection play crucial roles in shaping offspring development and health.

The topic of age (older parents), metabolic status and IVF has been extensively studied in human and indicate substantial intergenerational effects (Kaltsas et al., 2024; Lane et al., 2015) but this is outside of the focus of this review.

## DNA methylation

**Figure 1:**
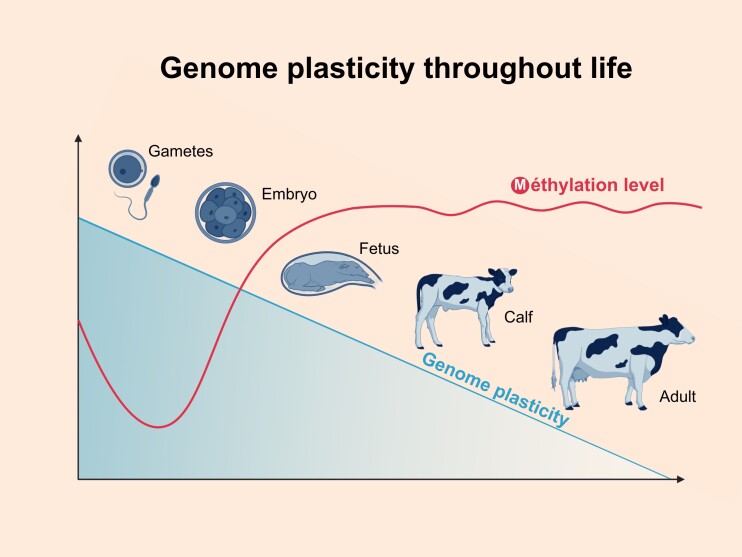
The majority of DNA methylation changes occurs during the cellular ontogeny and the plasticity of the program is decreasing with age although the absolute level of methylation does not change much in the tissues for which we have information like blood cells level.

In mammals, DNA methylation serves as a chemical modification of one of the four nucleotide bases in the DNA sequence, specifically within a CpG context. The majority of genomic methylation remains stable throughout an individual's life (Figure 1), with only minor changes occurring during specific cellular contexts. Two notable exceptions to this stability arise during embryonic development: the pre-implantation and primordial germ cell (PGC) stages. During these phases, most CpG sites undergo global demethylation followed by re-methylation (Wang et al., 2014).

However, in mammals, a small subset of DNA methylation marks is safeguarded from the second wave of genome-wide reprogramming. This protection is not only essential for normal embryogenesis but also enables the inheritance of traits acquired by parents to future generations (Jiang et al., 2018). The dynamic landscape of the DNA methylome plays critical regulatory roles in gene expression, genomic imprinting, embryo development, and chromosome structure (Smith and Meissner, 2013).

## Histone modifications

Histones are proteins that bind to DNA and regulate the accessibility of the transcription machinery necessary for gene expression. During spermatogenesis and maturation, histones are gradually replaced by protamine, which helps to condense the DNA in the sperm head. Nevertheless, approximately 1–15% of histones are retained, particularly in DNA regions that remain accessible shortly after fertilization (Siklenka et al., 2015). Recent research has characterized open-histone-rich chromatin regions in bull sperm and their association with both sperm fertility and the fertility of their offspring (Vargas et al., 2024).

In contrast, this histone replacement does not occur in oocytes. Instead, histones in oocytes undergo various spatial and temporal post-translational modifications, such as acetylation, methylation, and phosphorylation. These modifications are critical for oocyte maturation and play a vital role in the maternal-to-zygotic transition that occurs following fertilization (Xia et al., 2019).

## Non-coding RNAs

Non-coding RNAs (ncRNAs), such as microRNAs (miRNAs), are transcripts that are not translated into proteins. These molecules play crucial roles in regulating a wide array of biological activities, including cellular proliferation, differentiation, apoptosis, metabolism, and chromosomal remodeling. Importantly, ncRNAs derived from both parental gametes are essential for normal embryonic development (McJunkin, 2018). Recently, our laboratory has investigated the miRNA components in oocytes (Mondou et al., 2012), and sperm (Wu et al., 2020b), highlighting their association with gamete quality and age.

## DNA methylation patterns across developmental stages

There are two waves of cytosine demethylation. The first wave occurs in the gonads, where the primordial germ cell DNA undergoes an almost complete demethylation process. The second wave happens just after fertilization, and this genome-wide demethylation is closely related to embryonic genome activation (EGA) in bovine, with the major reduction of DNA methylation completed by the 8-cell stage (Jiang et al., 2018). However, a small portion of DNA methylation is maintained during the global reprogramming after fertilization, including imprinted genes, which could become a legacy of the parental environment effects revealed later in the offspring phenotype (Jiang et al., 2018).

The effects of maternal nutritional status, age, stress, lifestyle, disease, and other factors have been reported to be transmitted to the next generations (Perez and Lehner, 2019). However, research on paternal non-genetic effects has been long neglected compared to the numerous studies undertaken on the maternal side. Benefitting from studies identifying the molecules that sperm transfers during fertilization, paternal epigenetic influences are now gaining more and more attention (Lismer and Kimmins, 2023)

## Non-genetic maternal effects

The condition of the mother has a profound impact on offspring across generations through epigenetic modifications in humans. Epidemiological studies have demonstrated that the F2 generations of mothers who experienced famine periods had a higher tendency toward metabolic disorders (Aiken et al., 2016). Maternal age is another factor that can influence the developmental outcomes of progeny.

With the objective of characterizing the effect of female age on embryos, we performed transcriptomic and epigenetic studies using IVF and bovine female donors at ages of 8-11 and 14 months with the same animals being re-used as their own controls. It is important to note that the oocyte number is greater at an earlier age, but the embryo quality is lower (Morin-Doré et al., 2017; Landry et al., 2016).

At the transcriptomic level, age-related contrast analysis (8 vs. 14 months and 11 vs. 14 months) revealed more than 200 differentially expressed genes (DEGs) in blastocysts for each contrast. The analysis of the molecular and biological functions of the differences in gene expression suggests a metabolic cause to explain the differences observed between embryos from immature and adult subjects. The mTOR and PPAR pathways, as well as the NRF2-mediated oxidative stress response pathways, were affected by donor age. Therefore, the main differences between embryos produced at peri-pubertal ages seem to be associated with metabolic genes, resulting in a more pronounce effect of in vitro conditions on blastocysts from younger heifers (Morin-Doré et al., 2017).

For the epigenetic comparison, age-related contrast analysis identified 5,787 and 3,658 DNA differentially methylated regions (DMRs) in blastocysts from heifers at 8 vs 14 and 11 vs 14 months of age, respectively. For both contrasts, the DMRs were distributed non-randomly in the different DNA regions. The DNA from embryos from 8 month-old donors was hypermethylated, while the DNA from embryos from 11 month-old donors displayed an intermediate phenotype. According to Ingenuity Pathway Analysis, the upstream regulator genes TP53, TGFβ1, TNF, and HNF4α influenced the expression of methylation sensitive targets, which were hypermethylated in embryos from younger donors (Morin‐Doré et al., 2020).

Others have observed that prenatal maternal conditions were significantly correlated with the daughters’ fertility and milk production in bovines. For example, daughters of dams that were younger at first calving produced more first-lactation daily milk, had higher body condition scores (BCS), but experienced difficulties conceiving (Banos et al., 2007). Meanwhile, dams with higher BCS tended to give birth to calves with higher BCS, with lower return rates, but slightly lower daily milk yields (Banos et al., 2007). The maternal metabolic status can therefore influence production and reproductive traits of subsequent generations through non-genetic pathways.

Recently, the influence of the maternal metabolic environment was studied by collecting embryos from dairy cows experiencing different levels of negative energy balance (NEB) at conception and analyzing their transcriptome and epigenome using microarrays (Chaput and Sirard, 2019). Transcriptomic data highlighted that the most significantly affected pathways were related to metabolism, such as protein synthesis (EIF2 Signaling and eIF4, translation factors), mitochondrial function (oxidative phosphorylation and mitochondrial dysfunction), and metabolism (Sirtuin signaling and mTOR signaling) (Chaput and Sirard, 2019).

Using a DNA methylation microarray, 462 DMRs (differentially methylated regions) were identified between embryos from cows in high and low NEB, with many of them being located in gene regions, including introns, exons, proximal promoters, promoters, and distal promoters (Chaput and Sirard, 2019). Most of these genic regions, except exons, were hypermethylated in embryos from cows experiencing severe NEB (Chaput and Sirard, 2019). Functional analysis of DMRs located in gene regions was consistent with the transcriptomic results and pointed toward metabolic pathways (AMPK signaling and mTOR signaling), which are significantly affected by maternal energy deficits (Chaput and Sirard, 2019). These DMRs were retained at the blastocyst stage, thus it is highly possible that they could affect the phenotype of the offspring intergenerationally.

The DNA methylation analysis of blood from eight calves produced from high and low BHB mothers resulted in 1,675 DMRs (p < 0.05), indicating a postnatal legacy. This study highlighted how embryos interact with the maternal environment and the potential for intergenerationally inherited phenotype transmission through epigenetic modifications (Zhang et al., 2021).

## Non-genetic paternal effects

Many studies on paternal epigenetic effects on offspring were conducted using the mouse model. The effects of metabolic disorders in male mice (Wei et al., 2014) can be inter-generationally inherited by disturbing DNA methylation levels in sperm. Specific ncRNAs in mature spermatozoa, apparently acquired during transit in the epididymis, are believed to mediate paternal diet-induced metabolic disorders in offspring (Sharma et al., 2018). Multiple epigenetic factors conveyed by sperm are affected by the environmental or physical conditions of bulls. In bovine, age-related DMRs were observed in spermatozoa (Lambert et al., 2018), and around 20% of differentially methylated cytosines were retained in blastocysts (Lambert et al., 2018; Wu et al., 2020a). Among the genes mapped with these CpGs, some were involved in spermatogenesis (FKBP6) and embryonic preimplantation development (AKT2).

As mentioned above, different DNA methylation patterns were observed in sperm collected from the same bulls at different pubertal stages (Lambert et al., 2018). Approximately 69% of the DMRs were in genic regions, including one associated with the paternally imprinted gene MEST (mesoderm specific transcript). Methylation levels of most DMRs were higher with increasing age(Takeda et al., 2019). Network analysis of these DMRs revealed that sperm function-related pathways, such as PKA signaling, sperm motility, calcium signaling, and protein G signaling pathways, were significantly affected by paternal age (Lambert et al., 2018). Hence, although young bulls can produce functional semen, epigenetic factors transmitted by spermatozoa could potentially impact embryo or offspring development.

To further study paternal age effects on embryos, blastocysts were produced by IVF with spermatozoa from the same bulls at different pubertal periods (10, 12, and 16 months, by natural collection) and oocytes from several matched (the same cow for each individual bull) adult cows (Wu et al., 2020a). Transcriptomic and epigenetic analyses were performed on four pairs, where the only difference between embryos was the age of the bull. The results revealed elevated mitochondrial dysfunction, suppressed oxidative phosphorylation, and reduced protein synthesis in blastocysts generated from younger bulls, suggesting a low energy status in these blastocysts (Wu et al., 2020a). Moreover, the affected metabolic and sperm function pathways observed in blastocysts were consistent with the sperm studies mentioned above (Lambert et al., 2018), suggesting paternal age effects on embryos mediated by epigenetic factors in sperm.

We then tested the miRNA content in the same bulls at different ages (Wu et al., 2020b). We found significant differential expression analysis for the four bulls compared to themselves, with two miRNAs significantly overexpressed and four miRNAs underexpressed in spermatozoa of bulls at 10 months compared to 16 months of age. Similarly, three miRNAs and one tsRNA were upregulated, while five miRNAs were downregulated at 12 months compared to 16 months of age. After filtering out the small non-coding RNAs known to be present in maternal gametes (Mondou et al., 2012; Orozco‐Lucero et al., 2014), ten differentially expressed miRNAs were selected from the two contrasts and revealed similar targets (genes) as our study on DNA methylation (Wu et al., 2020b), showing a highly convergent strategy to influence the embryonic phenotype.

Then, we evaluated the histone retention profiles of bulls according to age. We used ATAC-seq on sperm collected from the same six bulls at the ages of 10 and 16 months. The major proportion of genes were more accessible in sperm collected when bulls were at the pre-pubertal stage, indicating histone-to-protamine transition deficiency, potentially due to the immature reproductive system in young bulls. A total of 195 genes were identified to have different accessibility in sperm collected from the same bulls when they were at pre-pubertal and post-pubertal ages. GO annotation of these genes demonstrated that protein ubiquitination, mitosis, cytoskeleton, and transcription were the most significantly affected cellular activities by paternal younger age (Wu, 2024). To investigate the potential effects of these genes in early embryos, we compared these genes in 2-cell embryos for further canonical pathway analysis by DAVID. Sixty-six DAGs were expressed in 2-cell embryos, and seven of them were related to 2-cell competence. Similar results were observed in DAGs expressed in 2-cell embryos, with enrichment in ubiquitin modification, transcription and peptide metabolic process. Positive regulation of chemokine production, response to hypoxia, and cytosol were enriched from the DAGs related to 2-cell competence. To assess whether these early-stage effects could impact later embryo development stages, we used upstream regulators of our previously reported transcriptomic data of blastocysts. We found two genes (Rarb and Hmga1) having different accessibility and capable of regulating their downstream targeted genes in the blastocyst stage (Wu et al., 2020a). Downstream targets of these two regulators revealed that basement membrane organization, protein heterodimerization, pre-miRNA transcription, and cell differentiation were enriched, indicating that paternal age could influence the gene expression pattern in blastocysts by altering the accessibility of several key genes.

In addition, the paternal metabolic status significantly influences semen quality. Although epigenetic studies related to the metabolic status of bulls are still missing, DNA methylation and histone modification patterns were both associated with bull fertility (Ugur et al., 2019). Functional annotation of these alterations indicated that they might be involved in spermatogenesis and embryo development (Ugur et al., 2019).

## Effects of *IVF*

Bovine assisted reproduction technology (ART) hinges on the combination of six techniques: ovarian stimulation, in vitro maturation (IVM), in vitro fertilization (IVF), in vitro embryo culture (IVC), embryo transfer (ET), and embryo freezing that when associated with genomic selection greatly accelerate genetic gains. With these techniques comes the potential to shorten e generation interval by using ovarian stimulation on prepubertal heifers (Sirard, 2018). The extensive use of ART has been linked to a higher incidence of several developmental disorders. Each aspect of the culture medium, including the physicochemical, oxidative, and energetic conditions, has profound effects on embryo development, and these effects could be maintained into adulthood or even subsequent generations (Calle et al., 2012) The presence of nutrients in concentrations that mimic in vivo conditions is fundamental to early embryogenesis, as demonstrated by the deleterious effects on embryo development of high concentrations of glucose and lipids in the culture medium (Cagnone and Sirard, 2014). Based on the metabolic pathways affected by culture conditions, embryos can either enhance a Warburg-like effect to adapt to minor stresses or induce apoptosis under severe stress. As a result, even though blastocyst rates may not be significantly impacted in modified media, embryo loss rates after transfer may be higher compared to embryos cultured in control conditions. Moreover, mitochondrial dysfunction is involved in the embryonic response to IVC stresses, and this may impact the mitochondria’s main role as energy factories, as well as the production of acetyl-CoA and methyl groups associated with one-carbon metabolism, which controls histone acetylation and DNA methylation. Thus, suboptimal IVC conditions do disturb the embryonic epigenome, which could contribute to the altered phenotype sometimes observed in offspring (Cagnone and Sirard, 2016; Lafontaine et al., 2023a).

## Postnatal phenotypes

Assisted Reproduction Technologies (ART) make it possible for a donor cow to produce multiple offspring, and these techniques can be applied to heifers only a few months after birth. Multiple Ovulation Embryo Transfer (MOET) can then be performed every 6-8 weeks. However, epigenetic changes in gametes, embryos, conceptuses, and newborns linked to ART-related alterations in the peri-conceptional conditions have been documented in several mammalian species. Multiple studies have reported on the long-term impact of ART. For example, IVF children presented higher systolic and diastolic blood pressure compared to their naturally conceived counterparts, as well as glucose metabolism impairment (Chen and Heilbronn, 2017). However, it is important to mention that ART impacts do not necessarily affect every individual but are rather limited to a population of ‘outliers’, alluding to the fact that most individuals/animals are either unaffected by, or compensate somehow for, the adverse peri-conceptual conditions.

Available literature on the long-term impacts of ART in cattle remains surprisingly sparse considering the intense phenotyping taking place in the dairy industry. The dairy industry collects phenotype information to generate large amounts of data on individual animals and herds, and the advent of livestock ‘big data’ has opened the door for high precision retrospective longitudinal cohort studies. A few examples at the herd level include studies by Siqueira et al. (2017, 2020), where records from 1,252 calves and 831 cows were analyzed. This study showed that IVF calves had higher birth weights and that cows derived from reverse X-sorted semen-IVF had lower milk yields in their first lactation. Because of the variation induced by embryo recipients in MOET and IVF, an experimental design comparing MOET vs. IVF vs. AI can effectively eliminate this recipient factor from the AI-IVF contrast, allowing a more specific characterization of outcomes related to in vitro manipulations.

We recently performed a retrospective cohort study with the Dairy Herd Improvement (DHI) dataset provided by Lactanet (Sainte-Anne-de-Bellevue, QC, Canada), which contains information about Quebec’s enrolled herds of Holstein cows that had at least one lactation between 2012 and 2019 and were either generated by AI, ET, or IVF (Lafontaine et al., 2023a, b). From a large phenotypic database (2.5 million animals and 4.5 million lactations), we identified 304,163 AI-, 12,993 MOET-, and 732 IVF-conceived cows (the vast majority from Quebec IVF lab, Boviteq) from which we retrieved information for 576,448, 24,192, and 1,299 lactations, respectively. Statistical analyses were performed using mixed linear models in SAS 9.4 software (SAS Institute; Cary, NC, USA). The Lifetime Performance Index (LPI) of the cow’s parents was used to normalize for genetic potential across animals.

When accounting for the higher LPI of MOET and IVF animals in the models, we found no statistical difference between the conception methods for the daughters’ milk production (beta hydroxy butyrate) across their first three lactations. The rate of LPI improvement of the IVF population during the 2012-2019 period was half the rate observed in the AI population. Preliminary fertility analysis revealed that MOET and IVF animals also scored one point lower on the daughter fertility index (Lafontaine et al., 2023a, b).

These results highlight the challenges of elite genetic improvement while attesting to the progress the industry has made to minimize epigenetic disturbance during embryo production. Still, additional work is required to ensure that IVF animals can achieve their full health and performance potential, notably by finding ways to avoid further increasing the energy allocation and metabolic programming for milk production at the expense of reproduction. However, gametes from young donors are suboptimal compared to those from adults as described above. Unfortunately, in the large epidemiological comparison of IVF vs ET and AI done in dairy cows, there was not enough very young donors to assess a significant effect of oocytes donor’s age on the observed quantifiable phenotypes (Lafontaine et al., 2023a).

## Conclusion

Parents provide more than purely genetic information to their offspring, and our studies indicate that the metabolic status, including that associated with younger age, is a key determinant of the epigenetic legacy that the embryo receives. The process is adaptive, and not all embryos will be impacted the same way by age or IVF. Postnatal analysis indicated that IVF has a visible influence on production and fertility in dairy cows but not enough young donor’s progeny were present in the epidemiologic analysis to conclude on age effect in F-1. This highlights the need for a better understanding of the molecular processes involved to minimize the possible negative consequences when optimizing culture conditions or managing young animals differently to achieve a metabolic status closer to optimal at gamete collection.
